# A Practical Alternative? KardiaMobile to Improve Uptake of ECGs on Psychiatric Wards

**DOI:** 10.1192/bjo.2025.10423

**Published:** 2025-06-20

**Authors:** Emily Pettifor, Faizaan Syed, Obumneme Chinweuba, Suresh Thapaliya, Mudasir Firdosi

**Affiliations:** 1 Kent and Medway NHS & Social Care Partnership Trust, Dartford, United Kingdom; 2 Kent and Medway NHS & Social Care Partnership Trust, Maidstone, United Kingdom; 3 Kent and Medway NHS & Social Care Partnership Trust, Canterbury, United Kingdom

## Abstract

**Aims:** ECGs on psychiatric wards are crucial for detecting cardiac side effects of psychotropics and identifying patients with underlying cardiac conditions. However, there can be variable patient uptake of standard 12-lead ECGs, which can lead to delays in initiating treatment and poor quality of physical health monitoring. KardiaMobile is a portable ECG device which can offer an alternative when 12-lead ECGs have been declined by patients, and was therefore explored as a way to improve ECG uptake. The aim was to reduce delays in ECG completion for patients admitted to inpatient settings and to explore the views of patients and staff around their experience of the device.

**Methods:** From September to October 2023, baseline data was collected retrospectively from one general adult psychiatric ward (A) and one psychiatric intensive care unit (B). This included the dates of patients’ admissions and the dates admission ECGs were completed. KardiaMobile devices were then introduced to wards as an alternative and in-person training sessions were delivered. Data was collected post-implementation of the devices from September 2024 to January 2025, recording use of KardiaMobile ECGs and dates of completion. Questionnaires were also used to collect patient and staff feedback.

**Results:** The metrics used were average delay to ECG completion (days) following inpatient admission and percentage of patients delayed by 3 or more days. On Ward A, baseline average delay to ECG completion was measured at 6.93 days and percentage of patients delayed by 3 or more days was 24.14%. Ward B recorded 12.77 days for average delay and 69.23% for delay by 3 or more days. Post-implementation data was collected until similar numbers of KardiaMobile ECGs were completed compared with baseline ECG data. There was insufficient uptake of KardiaMobile identified on Ward A for comparison, with patients reported to decline use. Ward B post-implementation recorded 0.5 days for average delay to KardiaMobile ECG completion and 7.14% of patients delayed by 3 or more days. Their feedback was overwhelmingly positive with respect to the use of the device and gave insight into why 12-lead ECGs had been declined.

**Conclusion:** There were identified reductions in delays on Ward B following introduction of KardiaMobile devices. Patients and staff expressed preference for the device due to its ease of use and convenience. Comparing data across the two separate wards has given insight into the possible challenges with use of KardiaMobile, as well as the significant benefits, suggesting potential applicability outside of inpatient settings.

